# Factor VIII haplotypes frequencies in Tunisian hemophiliacs A

**DOI:** 10.1186/1746-1596-6-54

**Published:** 2011-06-17

**Authors:** Hejer Elmahmoudi, Nejla Belhedi, Asma Jlizi, Kaouther Zahra, Balkis Meddeb, Amel Ben Ammar Elgaaied, Emna Gouider

**Affiliations:** 1 Laboratory of Genetics, Immunology and Human Pathologies, Faculty of Sciences of Tunis, University ElManar, Tunisia; 2 Hemophilia Treatment Centre, Aziza Othmana Hospital, University ElManar, Tunisia

## Abstract

**Background:**

The development of inhibitors against factor 8 (F8) is the most serious complication of replacement therapy with F8 in children with severe hemophilia. It was suggested that mismatched F8 replacement therapy may be a risk factor for the development of anti-factor F8 alloantibodies. Recently four single nucleotide polymorphisms (SNPs) encoding six distinct haplotypes, designated H1 through H6, were studied in different populations. Two SNPs are components of the A2 and C2 immunodominant-inhibitor epitopes.

The aim of this study is to determine the different types of haplotypes in relation with inhibitors developments and their frequencies in our Tunisian hemophiliac population.

**Materials and methods:**

95/116 Tunisian patients with hemophilia A undergoing treatment at Hemophilia Treatment Center, Aziza Othmana hospital, participate in this study. Among them only six patients develop inhibitors. The four SNPs were amplified and sequenced.

**Results and Discussion:**

In a total of 77 patients, we identified the H1, H2, H3 and the infrequent H5 haplotypes. The H1 and H2 haplotypes, which have the same amino acid sequence in the recombinant F8 molecules used clinically, are the most represented with the frequency of 0.763 and 0.157 respectively. This distribution is almost similar to that of Caucasians in which the frequencies are respectively 0.926 and 0.074, whereas it is 0.354 and 0.374 among Subsaharians. Four patients with inhibitors studied here have the H1 haplotype. For one patient who has a large deletion including the exon 10 we can't identify his haplotype. Theses frequencies may explain partially the low level of inhibitors in our patients.

## Introduction

Hemophilia A is a recessively inherited X-linked bleeding disorder which results from deficiency of factor VIII (F8). Treatment consists of substitution with plasma derived or recombinant F8 (rFVIII) [1]. F8 inhibitor is the most serious complication of replacement therapy with F8 in children with severe hemophilia. It remains unclear why it concerns only proportion of patients with hemophilia A. Several factors are reported: genetic, environmental, immunologic, treatments type... [2]. It was recently reported that several single-nucleotide polymorphisms (SNPs) identified in the F8 gene may play a role in the inhibitor development. Their incidence differs significantly in different ethnic groups [3]. Four non synonymous SNPs: G1679A (exon10), A2554G (exon14), C3951G (exon14) and A6940G (exon25) encoding respectively R484H, R776G, D1241E and M2238V [3,4]. The R484H and M2238V are components of the A2 and C2 immunodominant epitopes, respectively, which have been mapped to residues located at epitopes R484 to I508 and E2181 to V2243. The R776G and D1241E are located in the B domain [5,6]. The allelic combinations (haplotypes) of the four SNPs encode six distinct wild-type F8 proteins, which were designated H1 through H6. Two of them H1 and H2, which have the same amino acid sequences as respectively Kogenate^® ^and Recombinate^®^, the recombinant F8 molecules used clinically [7,8], were found in all studied populations with a high prevalence in Caucasians. The haplotypes H3, H4, and H5 were found only in Subsaharian populations and the haplotype H6 was found only in Chinese people [9].

In Tunisia recombinant F8 replacement therapy was recently introduced in 2008 for some patients. Patients were used to be treated with plasma derived factor. In order to identify the genetic background concerning the SNPs and the frequency of different haplotypes of our Tunisian hemophiliac A patients, we focused for the first time, on the R484H, R776G, D1241E and M2238V SNPs.

## Design and methods

### Patients

95 patients with hemophilia A undergoing treatment at Hemophilia Treatment Center, Aziza Othmana Hospital participate in this study. Each of the 95 enrolled patients provided a blood sample. Patients or their parents gave written informed consent for participation in this study and the research is carried out in accordance with the Helsinki Declaration.

### PCR/sequencing

Haplotype analysis using four amplicons of genomic F8 DNA that contain respectively the R484H, R776G, D1241E and M2238V SNPs were performed by the polymerase chain reaction (PCR) and sequenced to genotype the known non synonymous SNPs in order to identify the different haplotypes which characterize our Tunisian hemophiliac A patients. Haplotypes were constructed as a simple combination of the patient's non synonymous SNP alleles as a result for the FVIII hemizygoty.

## Result and discussion

The number of all identified Tunisian hemophiliacs A is 219 (Table 1) and 116 (53%) of them are treated in the Hemophilia Treatment Center of Aziza Othmana Hospital [10]. Among whom 95 were enrolled in the present study. Their age ranged between 2 and 51 years and they have been classified into three groups according to disease presentation 65 severe (68.48%), 26 moderate (27.36%) and 4 mild (4.21%).

**Table 1:** The Tunisian hemophilia A incidence in comparison to that of other Mediterranean countries according the World Federation of Hemophilia Report on the ANNUAL GLOBAL SURVEY 2007

Country	Haemophilia A Incidence	Number of inhabitants
Tunisia	219	10,383,577

Algeria	962	33,769,669

Egypt	3365	81,713,517

France	3618	64,057,790

Italy	2697	58,145,321

Greece	739	10,722,816

F8 haplotypes were established by sequencing four amplicons obtained from each 95 hemophiliac patients genomic DNA. We identified the H1, H2, H3 and the infrequent H5 haplotypes in our patients, the two others haplotypes H4 and H6 characteristic respectively of Subsaharian and Chinese populations are absent in our hemophiliac patients. One patient was excluded from the association analyses, since he could not be classified within either haplotype group because he has a lager deletion including the exon 10. After checking that related patients share the same haplotype, we exclude 18 patients who have a familial background and we selected only one patient from each family.

**Figure 1:**
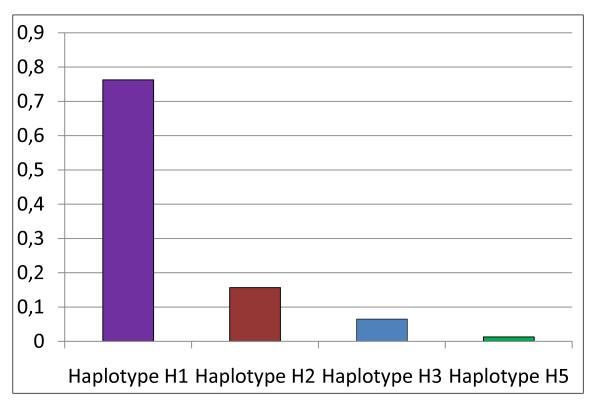
**Haplotype frequencies of factor VIII gene identified in Tunisian hemophiliac A**.

Hence we retained only 77 unrelated patients who were classified into 58 patients with H1 haplotype, 12 patients with H2 haplotype, 5 patients with H3 haplotype and 1 patient with H5 haplotype (Figure 1).

The H1 haplotype is the most frequent in our sample with a frequency of 0.763. This frequency is close to that described for Caucasian (0.926) and Chinese (0.846) populations and higher than that found in Subsaharian populations (0.354).

The H2 haplotype is present in our population with a frequency of 0.157 that is intermediate between that observed in Subsaharian populations (0.374) and that observed in Caucasian populations (0.074) and Chinese (0.077).

**Figure 2:**
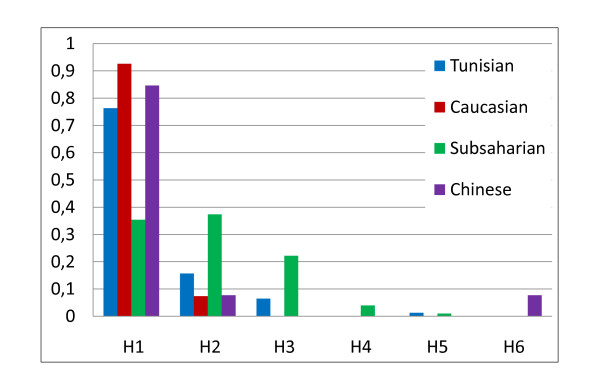
**Comparison of haplotype frequencies between Tunisian hemophiliac and others ethnic groups**.

For the H3 and H5 haplotypes, which are present in African populations [11] and absent in Caucasian and Chinese populations, we found the same frequency for H5 (0.013) and a lesser frequency (0.065) between our studied sample and Subsaharian populations. Our results as compared with other ethnic groups showed an intermediate position of the Tunisian sample between Caucasian and Subsaharian populations (Figure 2). This result is in agreement with previous studies indicating that Tunisian population is issued from a mixture with Eurasian and Subsaharian contributions [11,12].

Nowadays, inhibitor development in patients with severe hemophilia is a major complication in patient care because they render classical substitution therapy ineffective. F8 inhibitors occur at a frequency of 20-30% in severe hemophilia A and 3% in hemophilia B, respectively [2]. Several studies suggest that predisposing genetic factors, environmental factors and their interactions contribute to the inhibitor phenotype [13].

Only 7 patients with inhibitors were identified in the Hemophilia Treatment Center of Aziza Othmana Hospital.

If disease severity is related with the deleterious mutations in F8 gene responsible for hemophilia (gene deletion, truncated protein), inhibitor production depends on several parameters. Immunogenicity of heterologous F8 used for therapy is function of its structural differences with autologous F8.

The polymorphisms studied here will play a role in the inhibitors development since they are localized in regions recognized with inhibitors (domains A2 and C2) and will give an account for clinical data, since the inhibitors development frequency is higher in some ethnic groups [14,15].

The nature of the product used for therapy (cryoprecipity, rFVIII) and the time of exposure seem to influence allo immunization [15].

In conclusion, we were able to study the haplotypes for 4 polymorphic sites linked to F8 gene, to provide the basic information and better genetic characterization of Tunisian hemophiliac population. Meanwhile, determination of F8 gene haplotypes might be important to avoid or limit inhibitor production. Since study of F8 gene polymorphisms in the Africa is scarce, our results may be useful for SNPs studies of F8 gene of hemophiliac A in other neighboring countries, particularly in North Africa.

## Competing interests

The authors declare that they have no competing interests.

## Authors' contributions

HE designed the study and wrote the manuscript. NB and AJ interpreted the data and performed molecular experiments. BM and KZ participated in the sampling of clinical data. ABAE and EG performed critical reading of manuscript and supervision. All authors have read and approved the final manuscript.
